# The Weight of Responsibilities: Time in Responsibilities and in Food Preparation

**DOI:** 10.1016/j.cdnut.2026.109504

**Published:** 2026-08-01

**Authors:** Katelin M Alfaro Hudak, Pourya Valizadeh, Rodolfo M Nayga, Elizabeth F Racine

**Affiliations:** 1 Texas A&M AgriLife Research Center at El Paso, El Paso, TX, United States; 2 Department of Agricultural Economics, Texas A&M University, College Station, TX, United States; 3 Korea University, Seoul, South Korea; 4 Department of Nutrition, Texas A&M University, College Station, TX, United States

**Keywords:** time use, committed time, paid and unpaid work, time scarcity, meal preparation

## Abstract

**Background:**

The 2025–2030 Dietary Guidelines for Americans emphasize whole, minimally processed foods. However, the time required to prepare these foods may present a barrier. There is limited research on how other responsibilities shape food preparation activities.

**Objectives:**

The objective of this study was to identify the daily life activities that adults in the United States consider responsibilities and evaluate the association between the amount of time spent on responsibilities and food preparation activities.

**Methods:**

Using data from a national survey among United States adults, we identified which activities adults consider responsibilities. We used the American Time Use Survey and the Eating and Health Module data from 2014–2016 and 2022–2023 to examine the relationship between time spent on responsibilities and food preparation in the pooled sample and stratified by household composition.

**Results:**

Households with children spent the most time in responsibilities and food preparation, with single-parent households spending less time in food preparation than dual-parent households. The estimated association between time in responsibilities and food preparation was small but significant and negative in the pooled sample and across household composition subgroups.

**Conclusions:**

The marginal association between responsibilities and food preparation time is small, suggesting that households largely protect food preparation time as responsibilities increase. However, cumulative effects across realistic variation in responsibilities are nontrivial (representing ≤9% to 16% of mean food preparation time), and the relative inelasticity itself suggests that many households may already be operating near a minimum feasible level of food preparation time, raising questions about the potential for policies to encourage additional home cooking without simultaneously addressing structural time burdens. The findings provide policy-relevant evidence that time scarcity, beyond individual preferences, is strongly associated with household food preparation decisions, suggesting that it may operate as a meaningful constraint rather than merely reflecting preference differences.

## Introduction

The average American’s diet quality is far from ideal, entailing high public health and economic costs [[1], [2], [3], [4]]. Food prepared at home tends to be healthier than food away from home [5,6] and is one avenue through which families can potentially improve the quality of their diets. The 2025–2030 Dietary Guidelines for Americans emphasize foods that are whole or minimally processed. However, preparing food at home—especially whole, minimally processed—entails a nontrivial time cost, including grocery shopping, preparation, and cleanup. Several necessary and time-consuming responsibilities (e.g., work and childcare) can compete with the time allocated to food preparation. Adults are likely to organize their time to meet their perceived responsibilities. As their responsibilities increase (e.g., raising a child), their need for time management increases. They may realize they must prioritize some responsibilities over others, and, as a result, they may not have time to complete them all.

Although the importance of prioritizing responsibilities is generally appreciated, limited research has examined which daily life activities adults in the United States consider responsibilities and how time spent in responsibilities is associated with time spent in food preparation. There is evidence that certain households, such as single-headed households and those with young children [7,8], have less discretionary time, indicating they spend more time on responsibilities. Thus, food preparation may not be prioritized.

Previous research has classified daily life activities into 4 (necessary, contracted, committed activities, and leisure) [9,10] or 3 categories (necessary, committed, and discretionary) [7,[11], [12], [13]]. To our knowledge, these classifications have not been evaluated empirically, and it is unclear whether they accurately reflect the perspectives of United States adults. Also, limited research has examined the relationship between time spent in responsibilities and food preparation, and differences by household composition.

To address these gaps, this study first identifies the daily life activities that United States adults consider responsibilities by fielding a nationally representative survey. This objective is exploratory; thus, we do not posit any hypotheses. We then evaluate whether competing nonfood-related responsibilities (identified in the first objective) crowd out time spent in food preparation activities, using data from the American Time Use Survey (ATUS). There is evidence that time constraints are associated with changes in eating patterns [12,14]. Thus, we hypothesize that greater time spent in responsibilities is associated with less time spent in food preparation.

By identifying which activities are perceived as unavoidable responsibilities and how they constrain food preparation time, this study provides evidence for policymakers to design nutrition and time-use interventions that reflect households’ real-world time constraints, particularly for families with children. Ultimately, these insights can inform public health strategies that move beyond information provision to address structural time barriers shaping dietary behaviors and related health outcomes.

## Methods

### Data

#### Responsibilities survey

To identify which activities United States adults consider responsibilities, we fielded a national survey. The target population was the general population of United States adults (18 y +). We aimed for an approximately even split between households with *1*) young children aged 0 to 4 y, *2*) children aged 5 to 12 y, *3*) adolescent children aged 13 to 17 y, and *4*) no children.

To assess which activities respondents considered obligatory rather than discretionary, we asked respondents to identify which activities they considered to be responsibilities for adults in general. Responsibilities were defined as an activity that one is required to do as part of a job, role, or legal obligation, and respondents were instructed to think of roles broadly (e.g., parent, friend, and member of society). This approach is consistent with prior research asking respondents to categorize activities as obligations within specified social roles [15,16]. For example, Finch and Mason [16] used vignettes and qualitative interviews to explore how British adults reason about what counts as a family responsibility. Hamilton [15] investigated how people attribute responsibility more broadly, distinguishing between responsibility as obligation, causality, and accountability. Our approach differs in that we asked respondents to make a binary categorization (responsibility or not) across a range of everyday activities not limited to a single role domain, and we provided an explicit definition to anchor respondents’ judgments. We pilot-tested the survey, including the responsibilities identification item, with 31 participants who subsequently completed a feedback questionnaire asking them to identify any unclear or ambiguous survey items. None flagged the responsibilities question. We refined the survey after considering participant feedback on other survey items. The survey also collected data on participant demographics and household characteristics.

Once finalized, we fielded the survey in the fall of 2024 and recruited respondents via Lucid, an online survey panel that uses quota sampling to provide samples that mirror national estimates on multiple demographic factors, including age, ethnicity, and region [17]. Participants affirmed their consent to participate.

#### Responsibilities survey measures

The primary measure of interest was whether respondents considered an activity to be a responsibility for adults in general. For these activities, we drew on the ATUS survey activity lexicon [18]. Response options were binary.

To explore how parents with children aged <18 y potentially think differently about responsibilities, we collected data on household composition: total household size, whether the respondent had children <18 y in their household, the number of children they had, and the ages of their children. We categorized the ages of children as 0 to 4 y, 5 to 12 y, and 13 to 17 y. We also collected data on participant sociodemographic characteristics, including sex, age, educational attainment, race and ethnicity, marital status, employment status, hours worked per week, household income, and participation in nutrition assistance programs [school meals, the Supplemental Nutrition Assistance Program (SNAP), and the Special Supplemental Nutrition Program for Women, Infants, and Children (WIC)].

#### ATUS

To estimate the association between the amount of time spent on responsibilities and time spent in food preparation, we use time-use diaries and questionnaire data from the 2014–2016 and 2022–2023 ATUS and the Eating and Health Module (EHM), pooled across years. The EHM is a semiregular module that includes questions on respondents’ eating patterns, participation in food and nutrition assistance programs, and grocery shopping and meal preparation behaviors [19]. The ATUS is a nationally representative, continuous survey sponsored by the Bureau of Labor Statistics and conducted by the United States Census Bureau. ATUS respondents are randomly selected from households that completed their last month of participation in the Current Population Survey. ATUS respondents are interviewed about how they spent their time on the previous day. Participant demographics are also included in the ATUS data. Sociodemographic and time use data are from the main ATUS survey. The EHM is a semiregular module that has been included with the ATUS interviews to collect data on time use patterns, nutrition, eating patterns, meal preparation activities, and nutrition assistance programs. Data on SNAP participation and whether the respondent is primarily or partially responsible for meal preparation in their household come from the EHM. We use data from the 2 most recent collection periods, 2014–2016 and 2022–2023. We restricted the analysis to respondents aged ≥18 y who participated in the EHM, non-holiday diary days (98.5%), and those reporting that they were primarily (62.7%) or partially (18.2%) responsible for household food preparation.

#### ATUS measures

Our outcome is time spent in food preparation activities (hereafter, food preparation), coding the following activities as food preparation according to previous literature: food and drink preparation, food presentation, kitchen and food cleanup, grocery shopping, and related travel [20,21].

Our primary explanatory variable is time spent in nonfood*-related* responsibilities. To construct this variable, we use results from the responsibilities survey and prior literature to categorize activities as responsibilities. Specifically, we categorize an activity as a responsibility if a majority (>50%) of both respondents with children and respondents without children independently classified it as a responsibility. For the one borderline activity (relaxing and thinking), we use the designation employed by Kalenkoski et al. [7], who categorized relaxing and thinking as discretionary rather than necessary or committed activities (analogous to our nonfood-related responsibilities variable). We note that although ∼92% of respondents categorized meal and snack preparation as a responsibility, these activities are excluded from the explanatory variable because food preparation is captured in our outcome variable. We calculate the total time (in minutes) spent in activities classified as nonfood-related responsibilities and food preparation activities.

We controlled for individual and household characteristics that may influence time in food preparation and nonfood-related responsibilities: sex [22,23], age [20,23], race/ethnicity [20,23], educational attainment [23], marital status [23], income-to-poverty ratio [20,22,23], SNAP participation [22], employment status [23,24], hours worked per week [24], household size [6,23], presence of a child or adolescent and their ages (0–4 y, 5–12 y, or 13–17 y) [20,24], and single-parent households [23]. We used information on time diary day of the week, survey year, state of residence, and whether the respondent was primarily or partially responsible for the household food preparation. We used EHM sample weights and clustered the SEs by state of residence.

### Empirical model and analyses

#### Online responsibilities survey

To identify which activities participants consider to be responsibilities for adults in general, we calculate summary statistics of the proportion of respondents (hereafter, households) who categorized each activity as a responsibility. We examine chi-square test statistics to compare differences among households with and without children.

#### ATUS

We calculate summary statistics of ATUS respondent sociodemographic and household characteristics and examine chi-square test statistics to compare differences among households with and without children.

Some respondents do not spend any time in food preparation on the diary day (36.4% of adults in 2014–2016 and 2022–2023). Therefore, because distinct processes may shape the probability that a respondent will engage in any food preparation and the amount of time spent in food preparation, we employ a double-hurdle model [25], which models both the decision to engage in food preparation and the duration of time spent on food preparation, as follows:

(1) $$F_{i1}^{*}=Z_{i}\beta +\delta_{t}+\gamma_{d}+\theta_{s}+\varepsilon_{i}\;\text{engagement}\;\text{decision}$$

(2) $$F_{i2}^{*}=\Pi_{i}\lambda +\delta_{t}+\gamma_{d}+\theta_{s}+v_{i}\;\text{duration}\;\text{decision}$$

(3) $$F_{i}=\Pi_{i}\lambda +\delta_{t}+\gamma_{d}+\theta_{s}+v_{i}\;\text{if}\;F_{i1}^{*}>0\;\text{and}\;F_{i2}^{*}>0$$

(4) $$F_{i}=0\;\text{otherwise}$$

where $F_{i1}^{*}$ reflects the decision to engage in food preparation, $F_{i2}^{*}$reflects the duration of time spent on food preparation, $F_{i}$ reflects the actual amount of time spent, and $Z_{i}$ is the vector of variables that shape the decision to engage in food preparation—including the time spent in nonfood-related responsibilities. $\Pi_{i}$is the vector of variables that influence the amount of time spent on food preparation, including time in nonfood-related responsibilities, $\delta_{t}$denotes survey year fixed effects, $\gamma_{d}$ denotes diary day-of-week fixed effects, $\theta_{s}$ denotes state-of-residence fixed effects, and $\varepsilon_{i}$ and $v_{i}$ are the stochastic error terms.

We are also interested in potential heterogeneity across household composition subgroups. To assess household composition as potential modifiers, we iteratively tested interaction terms between nonfood-related responsibilities and indicators for the following 5 groups: *1*) households without children; *2*) households with children aged 0 to 4 y; *3*) households with children aged 5 to 12 y; *4*) households with adolescent children aged 13 to 17 y; and *5*) single-parent households. Groups of households with children of different ages are non-mutually exclusive. For example, households with a newborn and a 5-y-old would be included in groups 1 and 2 (Supplemental Table 1 for summary statistics of the overlap between households with children).

The main analysis included only those reporting that they were at least partially responsible for household food preparation. We examined whether our results were sensitive to including those who reported never being responsible for meal preparation. We also assessed whether our results were robust to an alternative threshold for an activity in the responsibilities survey being identified as a responsibility. That is, rather than a threshold of 50%, we tested using a threshold of 80%. Finally, we assessed risk of omitted variable bias by using the method developed by Oster [26], which evaluates the stability of coefficients in the presence of unobservable variables while accounting for the amount of variance in the outcome explained by the observed variables. However, Oster developed this method using linear models. Therefore, we ran ordinary least squares models that included the same control variables and applied Oster’s method to test the robustness of our results. We conducted all analyses using Stata 18.5 [27]. The Texas A&M Institutional Review Board reviewed the study protocol and deemed it exempt (responsibilities survey) or not human subject research (ATUS and EHM).

## Results

### Responsibilities survey

In the fall of 2024, 3999 participants completed the online survey, 75% (*n* = 3019) of which had children in their household (Table 1). The majority of survey respondents were female (64.7%), non-Hispanic White (61.8%), and had a high school degree (36.0%) or some college (22.5%) as their highest level of education. Half (49.8%) of the respondents worked full time and a quarter (25.0%) were unemployed. Over one-third of the sample reported receiving SNAP benefits (42.8%), and among those with children attending a school that offered school meals, nearly half (45.1%) received free meals.

**TABLE 1:** Responsibilities survey respondents’ demographic and household characteristics

	Total		No children in the household (24%)^1^		Children in the household (75%)^1^		*P* value^2^
	*n* = 3999		*n* = 965		*n* = 3019		
	Column %	SE	Column %	SE	Column %	SE	
Age (y, mean)	43.7	0.2	55.4	0.5	40.0	0.2	0.000
Sex (male)	35.3	0.8	47.6	1.6	31.4	0.8	0.000
Race/ethnicity							0.000
NH White	61.8	0.8	70.6	1.5	59.0	0.9	
NH Black	20.1	0.6	16.1	1.2	21.3	0.7	
Hispanic	11.1	0.5	6.4	0.8	12.6	0.6	
NH other/multiracial	7.0	0.4	6.8	0.8	7.0	0.5	
Education							0.834
High school/GED	36.0	0.8	36.6	1.6	35.8	0.9	
Some college	22.5	0.7	22.9	1.4	22.4	0.8	
Associate degree	13.2	0.5	12.4	1.1	13.4	0.6	
Bachelor’s degree	18.6	0.6	19.0	1.3	18.5	0.7	
Graduate or professional degree +	9.7	0.5	9.0	0.9	9.9	0.5	
Married	48.0	0.8	37.1	1.6	51.5	0.9	0.000
Household size (mean)	3.4	0.0	2.0	0.0	3.9	0.0	0.000
Number of children (mean)	1.4	0.0	0.6	0.0	1.7	0.0	0.000
Employment status							0.000
Work full time	49.8	0.8	31.9	1.5	55.5	0.9	
Work part time	11.7	0.5	11.7	1.0	11.7	0.6	
Unemployed^3^	25.0	0.7	18.3	1.2	27.1	0.8	
Retired	12.2	0.5	37.4	1.6	4.2	0.4	
Student	1.3	0.2	0.6	0.3	1.5	0.2	
Hours worked per week among those working full time or part time (mean)^4^	37.5	0.2	35.8	0.6	37.9	0.2	0.000
Household income^5^							0.000
≤$24,999	24.1	0.7	29.3	1.5	22.5	0.8	
$25,000–$74,999	47.1	0.8	49.3	1.6	46.4	0.9	
$75,000+	28.7	0.7	21.3	1.3	31.1	0.8	
Has child 0–4 y	31.2	0.7	14.7	1.2	36.3	0.9	0.000
Has child 5–12 y	42.9	0.8	12.8	1.1	52.2	0.9	0.000
Has child 13–17 y	38.7	0.8	19.2	1.3	44.8	0.9	0.000
School meals price^6^							0.036
Full price	32.8	1.2	21.2	4.6	33.4	1.2	
Reduced price	22.2	1.1	31.2	5.2	21.7	1.1	
Free	45.1	1.3	47.5	5.6	44.9	1.3	
Received SNAP	42.8	0.8	29.2	1.5	47.2	0.9	0.000
Received WIC	18.8	0.6	5.3	0.7	23.1	0.8	0.000

Abbreviations: NH, non-Hispanic; SNAP, Supplemental Nutrition Assistance Program; WIC, Special Supplemental Nutrition Program for Women, Infants, and Children.

1 Because we are particularly interested in how parents with children aged <18 y think about responsibilities, we targeted ∼75% of the sample to be parents with children.

2 Tests of significance assessed differences in characteristics between respondents with children in the household and those with no household children. Chi-squared tests were used for categorical variables. Wald tests were used for continuous variables.

3 Unemployed include those who are unemployed but looking for work, not working and not looking for work, and those who do not work due to a disability or illness.

4 The denominator for weekly hours worked is respondents reporting working full time or part time.

5 Respondents were asked to indicate their household income level, including income such as wages/salaries, Social Security, or retirement benefits.

6 The denominator for the price paid for school meals is the number of respondents indicating that their child attends a K-12 school that serves school breakfast or lunch.

Respondents with children in the household differed significantly from those without children on most measures. For example, respondents with children were significantly younger (40 y vs. 55 y), more likely to work full time, and more likely to receive SNAP or WIC benefits.

Table 2 shows the percentage of survey respondents indicating a given activity is a responsibility for adults in general and differences between households with and without children. As expected, the majority of respondents (≥95%) agreed that working at a job for pay, paying household bills, handling household financial management, and physical care of children were responsibilities. Among the activities that were more ambiguous, most considered basic self-care such as sleeping (76.4%) and self-grooming (93.7%) as responsibilities, whereas few considered attending religious services (35.6%) or volunteering (36.5%) to be responsibilities. Interestingly, 92.1% identified preparing meals and snacks as a responsibility.

**TABLE 2:** Percentage of responsibilities survey respondents indicating an activity is a responsibility for adults in general (*n* = 3984)^1^

	Total		No children in the household		Children in the household		*P* value^2^
	Column %	SE	Column %	SE	Column %	SE	
Paying household bills	97.9	0.2	97.5	0.5	98.0	0.3	0.3830
Working at a job for pay	95.8	0.3	93.9	0.8	96.4	0.3	0.0010
Handling household financial management	95.6	0.3	95.0	0.7	95.8	0.4	0.3110
Grocery shopping	95.4	0.3	93.5	0.8	96.0	0.4	0.0010
Physical care for household children	95.1	0.3	90.5	0.9	96.6	0.3	0.0000
Doing laundry	94.5	0.4	93.0	0.8	95.0	0.4	0.0150
Providing or obtaining medical care for household children	94.2	0.4	91.3	0.9	95.1	0.4	0.0000
Self-grooming	93.7	0.4	91.1	0.9	94.5	0.4	0.0000
Cleaning kitchen	93.6	0.4	91.1	0.9	94.5	0.4	0.0000
Helping household children with homework	93.3	0.4	88.1	1.0	95.0	0.4	0.0000
Interior cleaning	93.2	0.4	89.7	1.0	94.3	0.4	0.0000
Preparing meals or snacks	92.1	0.4	88.3	1.0	93.3	0.5	0.0000
Shopping for items besides groceries, food, and gas	89.6	0.5	84.1	1.2	91.3	0.5	0.0000
Providing physical care for household adults	87.7	0.5	89.0	1.0	87.2	0.6	0.1390
Attending household children's events	86.8	0.5	81.1	1.3	88.6	0.6	0.0000
Engaging in household and personal organization	84.2	0.6	78.0	1.3	86.2	0.6	0.0000
Lawn, garden, and houseplant care	83.0	0.6	80.0	1.3	83.9	0.7	0.0050
Playing with household children	77.8	0.7	69.0	1.5	80.6	0.7	0.0000
Reading to a household child	76.6	0.7	70.4	1.5	78.6	0.7	0.0000
Sleeping	76.4	0.7	71.1	1.5	78.1	0.8	0.0000
Eating and drinking	76.2	0.7	73.9	1.4	76.9	0.8	0.0570
Relaxing or thinking	51.7	0.8	46.2	1.6	53.5	0.9	0.0000
Participating in sports, exercise, and athletic recreation	40.4	0.8	33.5	1.5	42.6	0.9	0.0000
Socializing and communicating with others	37.6	0.8	33.6	1.5	38.9	0.9	0.0030
Participating in volunteer activities	36.5	0.8	35.3	1.5	36.8	0.9	0.4000
Attending religious services	35.6	0.8	33.0	1.5	36.4	0.9	0.0490
Reading for personal interest	28.2	0.7	26.4	1.4	28.8	0.8	0.1560
Computer use for leisure	20.6	0.6	19.3	1.3	21.0	0.7	0.2400
Watching TV	19.0	0.6	18.3	1.2	19.1	0.7	0.5790
Playing games	17.0	0.6	15.2	1.2	17.5	0.7	0.0990

1 Fifteen respondents had missing data on whether they had children aged <18 y living in their household.

2 Tests of significance assessed differences in activity categorization between respondents with children in the household and those with no household children. Chi-squared tests were used for all activities.

There were significant differences in how households with children classified activities compared with those without children. To illustrate, households with children were more likely to categorize working at a job for pay, preparing meals and snacks, providing physical care for children, and engaging in interior cleaning as responsibilities, among other activities.

### ATUS time use patterns

The majority of ATUS respondents were non-Hispanic White (66.1%) and had a high school (28.9%) or bachelor’s (22.9%) degree as their highest level of education (Table 3). Half (50.9%) worked full time, and one-third (36.4%) were not working. Two-thirds (67.4%) of households did not have children. Among those who did, one-third (33.7%) had a child aged between 0 and 4 y, half (50.8%) had a child aged 5 to 12 y, and one-third (33.9%) had an adolescent child aged 13 to 17 y. Among households with children, 12.9% were single-parent households. Households with children differed significantly from those with no children on virtually all measures. For example, respondents with children were significantly younger (39.3 y vs. 53.0 y), were more likely to work full time, and were more likely to receive SNAP benefits (14.8% vs. 7.3%). In general, sociodemographic characteristics of the ATUS sample were qualitatively similar to the responsibilities survey sample.

**TABLE 3:** American Time Use Survey-Eating and Health Module respondents’ demographic and household characteristics^1^

	Total (*n* = 37,368)		No children in the household (*n* = 25,169)		Children in the household (*n* = 12,199)		*P* value^2^
	Column %	SE	Column %	SE	Column %	SE	
Age (y, mean)	48.6	0.1	53.0	0.2	39.3	0.2	0.0000
Sex (male)	40.5	0.3	43.2	0.4	34.9	0.6	0.0000
Race/ethnicity							0.0000
NH White	66.1	0.3	71.0	0.4	55.9	0.6	
NH Black	11.8	0.2	12.1	0.3	11.1	0.4	
NH Asian	5.0	0.2	4.3	0.2	6.4	0.3	
Hispanic	15.4	0.3	11.0	0.3	24.4	0.6	
NH other/multiracial	1.7	0.1	1.5	0.1	2.2	0.2	
Educational attainment							0.0000
< High school	8.8	0.2	7.5	0.2	11.7	0.5	
High school/GED	28.9	0.3	30.2	0.4	26.0	0.6	
Some college	15.6	0.3	15.9	0.3	14.9	0.4	
Associates degree	9.6	0.2	9.4	0.2	9.8	0.3	
Bachelor’s degree	22.9	0.3	23.0	0.4	22.5	0.5	
Graduate or professional degree +	14.3	0.2	13.9	0.3	15.0	0.4	
Married	53.3	0.4	45.4	0.4	69.8	0.6	0.0000
Employment status							0.0000
Work full time	50.9	0.3	46.8	0.4	59.4	0.6	
Work part time	12.7	0.2	11.8	0.3	14.5	0.4	
Not working^3^	36.4	0.3	41.4	0.4	26.1	0.6	
Usual weekly hours worked among those working full time or part time (mean)^4^	39.7	0.1	39.6	0.1	39.8	0.2	0.4668
Poverty–income ratio (mean)	3.7	0.0	4.0	0.0	3.0	0.0	0.0000
SNAP participation							0.0000
Receives SNAP	9.7	0.2	7.3	0.2	14.8	0.5	
Low-income (≤185% FPL) nonparticipant	19.1	0.3	17.8	0.3	21.7	0.6	
Higher-income (>185% FPL) nonparticipant	71.2	0.3	74.9	0.4	63.5	0.6	
Household size (mean)	2.7	0.0	2.0	0.0	4.2	0.0	0.0000
Number of household children (mean)	0.6	0.0	—	—	1.9	0.0	—
Has child 0–4 y	10.9	0.2	—	—	33.7	0.6	—
Has child 5–12 y	16.4	0.2	—	—	50.8	0.6	—
Has child 13–17 y	11.0	0.2	—	—	33.9	0.6	—
Dual parent	23.2	0.3	—	—	71.6	0.6	—
Single parent	4.2	0.1	—	—	12.9	0.3	—
Diary day of the week							0.6280
Sunday	14.6	0.2	14.4	0.2	15.0	0.3	
Monday	13.6	0.3	13.5	0.3	13.8	0.5	
Tuesday	14.5	0.3	14.4	0.3	14.9	0.5	
Wednesday	14.5	0.3	14.5	0.3	14.4	0.5	
Thursday	14.3	0.3	14.3	0.3	14.2	0.5	
Friday	14.2	0.3	14.4	0.3	13.7	0.5	
Saturday	14.3	0.2	14.4	0.2	14.0	0.3	

Abbreviations: FPL, federal poverty line; GED, General Educational Development; NH, non-Hispanic; SNAP, Supplemental Nutrition Assistance Program.

1 Data from 2014–2016 and 2022–2023 American Time Use Survey-Eating and Health Module. Sample includes respondents aged ≥18 y, those who reported being at least partially responsible for preparing meals in their households, and diary days that were not holidays.

2 Tests of significance assessed differences in characteristics between respondents with children in the household and those with no household children. Chi-squared tests were used for categorical variables. Wald tests were used for continuous variables.

3 Includes respondents who reported being unemployed, not in the labor force, or students.

4 The denominator for usual weekly hours worked is respondents reporting working full time or part time.

Table 4 presents summary statistics of time spent in nonfood-related responsibilities and food preparation in the pooled sample and by household composition. In all groups, 100% of respondents spent some time in nonfood-related responsibilities. Households with adolescent children (aged 13–17 y) spent the most time in nonfood-related responsibilities (mean: 1092 min, or 18.2 h), whereas households with no children spent the least amount (mean: 940 min, or 15.7 h). This pattern was also observed in the mean time spent in food preparation. Households with adolescent children spent the most time in food preparation (96 min, or 1.6 h), and households with no children spent the least time (75 min). Single-parent households spent 11 min less in food preparation than dual-parent households.

**TABLE 4:** Mean time (minutes per day) American Time Use Survey-Eating and Health Module respondents spent in select activities^1^

	Nonfood-related responsibilities^2^		Food preparation		
	Time in the activity^3^		Percentage spending time in activity (%)	Time in the activity^3^	
	Mean	SE		Mean	SE
Pooled sample	969.2	1.6	71.3	79.7	0.6
Households with no children	939.5	2.0	69.2	74.5	0.7
Households with children 0–4 y^4^	1062.6	3.5	78.7	90.9	1.7
Households with children 5–12 y^4^	1041.4	3.0	77.8	92.7	1.4
Households with children 13–17 y^4^	1091.9	4.3	75.8	95.8	2.0
Dual-parent households^5^	1043.8	2.6	76.7	92.5	1.2
Single-parent households^5^	1033.4	5.8	78.6	81.5	2.5

1 Data from 2014–2016 and 2022–2023 American Time Use Survey-Eating and Health Module. Sample includes respondents aged ≥18 y, those who reported being at least partially responsible for preparing meals in their households, and diary days that were not holidays.

2 Nonfood-related responsibilities comprise 7 activity categories: personal care (which includes sleeping), working and work-related activities, eating or drinking, household activities, caring for or spending time with children, helping household adults, and shopping (grocery shopping is not included in nonfood-related responsibilities but is included in food preparation). The percentage of respondents spending any time in nonfood-related responsibilities is not presented because 100% of respondents spent time in nonfood-related responsibilities.

3 Mean time is calculated for those who reported spending some time in each activity.

4 Households consist of both dual- and single-parent families.

5 Households consist of children of all ages, 0–17 y.

Table 5 presents the estimated associations of time spent on nonfood-related responsibilities and food preparation. In examining heterogeneity by household composition, all interaction terms were significant (*P* < 0.05). Therefore, in addition to the pooled sample, we stratified the analysis by household composition subgroups. In all models, we find that time spent on nonfood-related responsibilities is significantly and negatively associated with the decision to engage in food preparation and the amount of time spent on food preparation (*P* < 0.001). However, the estimated association is −0.049 in the fully adjusted model (column 2), indicating that an additional 10 min spent on nonfood-related responsibilities is associated with a half-minute reduction in food preparation time. We generally find larger effects for households with children. For example, the estimated effect size in households with adolescent children aged 13 to 17 y is double that in the pooled sample. The estimated association is smaller in single-headed households than in dual-parent households (−0.056 vs. −0.092, respectively), indicating that time spent in food preparation is less elastic for these households.

**TABLE 5:** Average marginal association of time in nonfood-related responsibilities on time in food preparation^1^

	1 Unadjusted	2 Fully adjusted	Household composition subgroups					
			3 Children 0–4	4 Children 5–12	5 Children 13–17	6 No children	7 Dual-parent households	8 Single-headed households
Responsibilities	−0.057∗∗∗	−0.049∗∗∗	−0.085∗∗∗	−0.085∗∗∗	−0.100∗∗∗	−0.043∗∗∗	−0.092∗∗∗	−0.056∗∗∗
SE	(0.003)	(0.003)	(0.009)	(0.007)	(0.010)	(0.003)	(0.007)	(0.011)
n	37,368	35,895	4115	6493	3723	24,247	8104	2284

Abbreviation: SNAP, Supplemental Nutrition Assistance Program.

^∗^*P* < 0.05; ∗∗*P* < 0.01; ∗∗∗*P* < 0.001.

1 Marginal associations estimated by double-hurdle models with robust SEs clustered on state of residence. Time in nonfood-related responsibilities and food preparation is in minutes. Time diary days that were holidays were excluded. Models include adults (≥18 y) who reported being primarily or partially responsible for meal preparation in the household. Time spent in nonfood-related responsibilities was modeled in the selection model and in the linear model. Control variables were the same in the selection and linear models and included respondent sex, age, age^2^, race/ethnicity, educational attainment, whether the respondent was married, poverty–income ratio, SNAP participation, employment status, usual number of hours worked per week, household size, indicators for household children aged 0–4 y, 5–12 y, and 13–17 y, and an indicator for single-parent households. Models 7 and 8 did not include a control variable for single-parent households. Models 2–8 included fixed effects for diary day of the week, survey year, and state of residence. Models stratified by whether there is a child in specific age ranges in the household (models 3–5) are non-mutually exclusive and may overlap. Models 6–8 are mutually exclusive.

Results were robust to including those who reported never being responsible for meal preparation (Supplemental Table 2). When conducting the analyses using the alternate definition of nonfood-related responsibilities (i.e., the 80% threshold), the same patterns of time use emerged, with a few differences. Once the activities of sleeping, eating and drinking, playing with children, and reading to children were removed, households with children aged 5 to 12 y (451 min) and single-parent households (450 min) spent the most time in nonfood-related responsibilities (Supplemental Table 3). The patterns observed in the regression results were similar to those seen in the main analysis (Supplemental Table 4). Finally, the results from Oster’s methods suggest that our findings are robust to omitted variables (Supplemental Table 5). The bounds do not include zero and the δ (which represents the relative degree of selection on unobservables compared with selection on observables) is >1, suggesting that our results are robust to omitted variables. Specifically, the δ is equal to 3.47, indicating that the effect of the omitted variables would have to be 3.5 times as large as the effect of the observed controls to fully attenuate the association between nonfood-related responsibilities and food preparation.

## Discussion

By fielding a national survey among United States adults, we identified which daily activities adults consider responsibilities. We applied these results to the ATUS to categorize activities as nonfood-related responsibilities and examined the association between time spent on nonfood-related responsibilities and on food preparation. We found that time spent on nonfood-related responsibilities is negatively associated with food preparation time, with differential associations by household composition.

Previous research categorized daily activities as necessary and committed activities [7,12], which are similar to our definition of nonfood-related responsibilities. Results of the responsibilities survey generally reflect the schema employed by Kalenkoski et al. [7,12], with few exceptions. Survey respondents indicated that both shopping and eating and drinking were responsibilities, whereas Kalenkoski et al. categorized them as discretionary.

Households with children of all age groups spent more time in both nonfood-related responsibilities and food preparation than households without children. This reflects Kalenkoski et al.’s [7] finding that individuals in households with children have less discretionary time than those without children. Regarding time spent in food preparation, a study using ATUS data from 2003 and 2016 found that in both time periods, households with children spent more time in food preparation than households without children [28].

The descriptive statistics are intended to compare mean time use across different household types, whereas the regression estimates quantify the marginal association between nonfood-related responsibilities and food preparation after conditioning on observed household characteristics. Thus, the positive unconditional association between nonfood-related responsibilities and food preparation observed across household types in Table 4 reflects compositional differences between households rather than the marginal effect of an additional minute in nonfood-related responsibilities. Households with children spend more time in nonfood-related responsibilities and in food preparation, but these are jointly driven by a third factor: the presence of children themselves. Children simultaneously generate nonfood-related responsibilities (e.g., supervision, physical care, help with homework) and necessitate greater food preparation (e.g., more mouths to feed, structured mealtimes, child-specific dietary needs).

The adjusted models examine the association between nonfood-related responsibilities and food preparation time among households with similar compositions. In this conditional comparison, the time scarcity mechanism emerges. In brief, the between-group pattern is positive because the factors that generate nonfood-related responsibilities also generate food preparation demands. The within-group (i.e., adjusted) pattern is negative because, holding those demand-generating factors constant, additional nonfood-related responsibility time competes with food preparation for a limited pool of available hours.

In related research, Kalenkoski and Hamrick [12] found that individuals who were time poor were 3% less likely to purchase fast food on an average day, which is contrary to what would be expected given the present findings. That is, if individuals who spend more time in nonfood-related responsibilities spend less time in food preparation, a logical hypothesis would be that those who spend more time in nonfood-related responsibilities are more likely to eat already prepared foods or fast foods. However, Kalenkoski and Hamrick [12] found the opposite.

We found evidence of heterogeneity across household composition by the presence of children. Estimated effect sizes were larger for households with children than those without children, indicating that for households with children, time spent in food preparation is more sensitive to the amount of time spent in nonfood-related responsibilities. Although not directly comparable, in an analysis of 2003–2004 ATUS data, Mancino and Newman [23] found that household composition was related to time spent in food preparation; the number of children and the presence of a young child (0–5 y) were both positively and significantly associated with the amount of time spent in food preparation.

Single-parent households spent approximately the same amount of time in nonfood-related responsibilities when compared with dual-parent households. This is in contrast to other research that found single-parent households have the highest rates of time poverty than any other category [7]. This difference in findings may be explained by alternative approaches to measuring time in responsibilities (continuous measure or threshold-based) or potential changes over time (2003–2006 ATUS vs. 2014–2016 and 2022–2023 ATUS). Interestingly, in regression analysis, the marginal association for single-parent households was nearly half that of dual-parent households, indicating a weaker relationship between nonfood-related responsibilities and food preparation time among single-parent households. These findings are similar to Cawley and Liu’s [24] results from an investigation of how maternal employment is associated with time spent in grocery shopping, cooking, eating with children, and other outcomes related to children’s diet-related health. They found that maternal employment (employed vs. not working) was associated with significant decreases in time spent in most outcomes examined, and the effects were larger for single-mother households than for households with a spouse or partner. Study authors posit that whereas partnered mothers can obtain help with childrearing-related tasks from their spouses or partner, single parents are unable to do so. The ATUS data only include time diary data for 1 person in the household, preventing us from investigating spousal cooperation.

Although there was a statistically significant, negative association between time in nonfood-related responsibilities and food preparation time, the magnitude of the estimated association was −0.049 in the pooled sample, which translates to a half-minute reduction in food preparation time for every 10 additional minutes spent on nonfood-related responsibilities. This represents 0.6% of mean daily food preparation time per 10-min increment. However, cumulative effects across realistic variation in nonfood-related responsibilities are nontrivial. For example, the difference in nonfood-related responsibilities between households without children (∼940 min) and those with adolescents (∼1092 min) is ∼152 min. Across this range, the cumulative effect would be ∼7.4 min (152 × 0.049), which is ∼9.3% of the mean food preparation time. That is, if there are 2 otherwise similar households, and 1 has 152 more minutes of nonfood-related responsibilities, the model predicts that the household would spend ∼7.4 min less on food preparation. For households with adolescents (estimated marginal association = −0.10), this cumulative difference would be even larger at ∼15 min, or 16% of their mean food preparation time of 95.8 min. This suggests that time constraints are already binding for many households.

Although our study does not directly measure diet quality, existing research provides relevant context regarding the findings’ plausible implications on dietary quality. Monsivais et al. [6] found that individuals spending more time on home food preparation had better dietary indicators, including higher fruit and vegetable consumption and lower likelihood of using convenience foods. Simple healthy meals typically require 15 to 30 min of active preparation (based on the USDA’s former “MyPlate” recipes [29] and recipes from former SNAP Education programs [30,31]). Meals based on whole, minimally processed ingredients typically require a more active preparation time than convenience or ultraprocessed alternatives [6,22]. A cumulative reduction of 7 to 15 min could thus represent the difference between preparing a meal from whole ingredients and relying on ultraprocessed convenience alternatives.

Overall, these findings highlight that time constraints operate as a meaningful, yet uneven, constraint on household food preparation, with particularly important implications for households with children. The relative inelasticity of food preparation time suggests that many households, particularly single-parent households, may already be operating near a minimum feasible level, limiting the effectiveness of policies that assume households can readily reallocate time toward home food preparation. Policies aimed at improving diet quality via home cooking and an emphasis on whole, minimally processed foods (a pathway supported by prior research linking home food preparation to healthier eating patterns [6]) may therefore be more effective if they reduce time burdens or provide structural supports, rather than relying solely on behavioral or informational interventions.

### Strengths and limitations

This study has several strengths, including being the first, to our knowledge, to empirically explore which activities United States adults consider responsibilities, applying these findings to a large, nationally representative time use survey to assess the connection between time in nonfood-related responsibilities and food preparation, and examining heterogeneity among household composition groups. Furthermore, our results were robust when using an alternate definition of nonfood-related responsibilities and when including those reporting not being responsible for meal preparation. Finally, when employing the method developed by Oster [26], results indicated the stability of coefficients in the presence of unobservable variables.

Still, several limitations should be considered. Time spent in nonfood-related responsibilities and food preparation is drawn from a fixed daily time budget and is likely shaped by common underlying constraints and preferences. Although we assessed potential bias from unobserved confounders, simultaneity can remain a concern because these time uses are jointly determined and may influence one another. Future research could strengthen causal inference by leveraging exogenous variation in household nonfood-related responsibilities. Relatedly, because of the fixed 24-h daily time budget, an increase in time spent on responsibilities necessarily reduces the amount of time available for other activities. Because the nonfood-related responsibilities measure includes sleep and personal care, the remaining time is allocated between food preparation and leisure. As a result, a negative association between responsibilities and food preparation is not mechanically implied, because reductions in available time may be absorbed through either food preparation or leisure. However, food preparation is small relative to leisure, and a proportional mechanical allocation of time would predict a larger negative association than we observe, suggesting that households partially protect food preparation time while disproportionately reducing leisure. Nonetheless, we cannot fully disentangle behavioral responses from the mechanical constraint imposed by the fixed time budget.

In addition, the samples from the responsibilities survey and from ATUS differed. We sought to obtain a representative sample in the responsibilities survey, and although it was representative on some measures (e.g., age, ethnicity, and region), SNAP participation differed, with 42.8% of the responsibilities survey sample reporting receiving SNAP benefits. By contrast, only 9.7% of the ATUS sample reported participating in SNAP. Also, the proportion of households with children differed by design, as we purposefully oversampled households with children in the responsibilities survey. These differences between samples pose a potential threat to the validity of our approach, because the classification of activities as responsibilities, which was derived from a sample with higher SNAP participation and a greater proportion of households with children, may not generalize to the broader population represented in the ATUS. If perceptions of what constitutes a responsibility vary systematically with economic circumstances or household composition, applying this classification to the ATUS sample could introduce measurement error or bias. To test the robustness of our findings with a sample that more closely resembles the ATUS sample in terms of the proportion of households with children, we weighted the data so that households with children had a weight of 0.44 and those without children had a weight of 2.68, resulting in a weighted proportion of 66% with no children and 34% with children. We categorized activities as responsibilities using this weighting schema and found no difference in the activities that would be categorized as responsibilities (results available on request). Future research should test the robustness of the responsibilities classification in samples that are nationally representative across these dimensions, including SNAP participation and other household characteristics. In addition, because we modeled nonfood-related responsibilities as an aggregate measure, we cannot identify which specific components (e.g., paid work, childcare, and sleep) most strongly compete with food preparation time. Future research decomposing nonfood-related responsibilities into subcategories could illuminate specific substitution patterns and inform more targeted interventions. Finally, ATUS data capture a single diary day per respondent, which may not reflect usual or longer-term patterns of time use.

### Conclusions

This study provides empirical evidence for classifying common daily activities as responsibilities, which can be used in future research. Although the online survey findings generally support the most recent classification schema, survey respondents’ categorizations differed for several activities, suggesting areas for future research. Our ATUS results indicate that time in nonfood-related responsibilities and food preparation are consistently and significantly associated in the pooled sample and across household types. However, the marginal association is small, suggesting that households largely protect food preparation time as responsibilities increase and have limited flexibility to adjust food preparation time in response to additional responsibilities. Heterogeneity across household composition subgroups further suggests that competing demands have a larger impact on food preparation among households with children. These findings reflect the importance of accounting for families’ time constraints when designing policies to improve dietary patterns by emphasizing home preparation of whole, minimally processed foods. However, this study examined food preparation time rather than diet quality directly, and the extent to which the observed time reductions translate into meaningful dietary changes warrants further investigation. Study findings point to the potential value of structural supports such as time-saving interventions (e.g., meal kits, access to prepared healthy foods, childcare supports) and workplace flexibility or scheduling policies that could enable healthier food preparation under real-world time constraints, particularly for families with children.

## Author contributions

The authors’ responsibilities were as follows – KMAH, PV, RMN, EFR: designed research; KMAH: conducted research, analyzed data, wrote the paper, and primarily responsible for final content; PV, RMN, EFR: reviewed and edited the paper; and all authors: read and approved the final manuscript.

## Data availability

Secondary data used in this study are publicly available from the Bureau of Labor Statistics (American Time Use Survey and Eating and Health Module; https://www.bls.gov/tus/). De-identified data from the primary survey are not yet publicly available because additional analyses are planned but will be made available from the corresponding author on reasonable request. The codebook and analytical code are available from the corresponding author on reasonable request.

## Declaration of Generative AI and AI-assisted technologies in the writing process

The authors declare that no generative AI or AI-assisted technologies were used in the writing of this manuscript.

## Funding

The authors reported no funding received for this study.

## Conflict of interest

EFR has received travel support and stipends for leading Working Groups organized by the Healthy Eating Research Program funded by the Robert Wood Johnson Foundation and the CDC Nutrition and Obesity Policy and Evaluation Network. KMAH, PV, and RMN have no known competing financial interests or personal relationships that could have appeared to influence the work reported in this paper.
