# Supplementary material for: The Weight of Responsibilities: Time in Responsibilities and in Food Preparation
Source: Curr Dev Nutr. 2026 Aug 1;10(9):109504. doi: 10.1016/j.cdnut.2026.109504 (PMC13524605; doi:10.1016/j.cdnut.2026.109504)
Supplement: Supplementary file 1 [file mmc1.docx]

# Supplemental Table 1. Overlap among households with children^†^

| Category | Frequency | Sample % | Population % |
| --- | --- | --- | --- |
| 0-4 only | 2,263 | 19.6 | 19.9 |
| 5-12 only | 3,341 | 28.9 | 25.3 |
| 13-17 only | 2,044 | 17.7 | 20.1 |
| 0-4 & 5-12 | 1,871 | 16.2 | 15.5 |
| 0-4 & 13-17 | 126 | 1.1 | 1.3 |
| 5-12 & 13-17 | 1,685 | 14.6 | 15.6 |
| All three ages | 236 | 2.0 | 2.2 |

^†^This table presents the unweighted frequencies and percents (sample %) and the weighted percents (population %) of households with children in different age groups.

# Supplemental Table 2. Average marginal association of time in responsibilities on time in food preparation: Including those who reported not being responsible for meal preparation^†^

|  |  |  | Household composition subgroups | | | | | |
| --- | --- | --- | --- | --- | --- | --- | --- | --- |
|  | 1 Unadjusted | 2 Fully adjusted | 3 No children | 4 Children 0-4 | 5 Children 5-12 | 6 Children 13-17 | 7 Dual-parent households | 8 Single-headed households |
| Responsibilities | -0.055*** | -0.042*** | -0.038*** | -0.059*** | -0.066*** | -0.077*** | -0.071*** | -0.048*** |
| SE | (0.003) | (0.003) | (0.003) | (0.008) | (0.006) | (0.008) | (0.006) | (0.010) |
| n | 37368 | 35895 | 24247 | 4115 | 6493 | 3723 | 8104 | 2284 |

*** p<.001, ** p<.01, * p<.05

^†^ Marginal associations estimated by double hurdle models with robust standard errors clustered on state of residence. Time diary days that were holidays were excluded. In the main analysis, models included adults (≥18 years) who reported being primarily or partially responsible for meal preparation in the household. However, 41% of adults in our sample who indicated not being responsible for meal preparation reported spending time in food preparation activities. This analysis includes adults who reported not being responsible for meal preparation and those who reported being primarily or partially responsible. Time spent in responsibilities was modeled in the selection model and in the linear model. Control variables were the same in the selection and linear models and included respondent sex, age, age^2^, race/ethnicity, educational attainment, whether the respondent was married, poverty-income ratio, SNAP participation, employment status, usual number of hours worked per week, household size, indicators for household children ages 0-4 years, 5-12 years, and 13-17 years, and an indicator for single-parent households. Models 7 and 8 did not include a control variable for single-parent households. Models 2-8 included fixed effects for diary day of the week, survey year, and state of residence.

# Supplemental Table 3. Time spent in responsibilities: Alternate definition of responsibilities^†^

|  |  | Time (minutes) in responsibilities | |
| --- | --- | --- | --- |
|  | % spending any time in activity | Mean | SE |
| Pooled sample | 97.2% | 378.5 | 1.7 |
| Households with no children | 96.6% | 352.1 | 2.1 |
| Households with children 0-4 y | 98.8% | 435.3 | 4.2 |
| Households with children 5-12 y | 98.9% | 451.4 | 3.4 |
| Households with children 13-17 y | 98.7% | 448.7 | 4.9 |
| Two-parent households | 98.7% | 446.5 | 3.1 |
| Single-parent households | 98.9% | 450.4 | 6.2 |

^†^ Alternate definition of *responsibilities* used a cut-off of 80% of respondents classifying an activity as a responsibility. With this stricter cut-off, sleeping, eating and drinking, playing with household children, and reading to a household child were no longer classified as activities. Respondents were largely consistent in which activities were identified as responsibilities. We explored alternate cut-offs (60%, 67%, 75%) and the responsibility classification remained unchanged, demonstrating that the classification is robust. The 80% threshold was selected because it was the lowest cutoff at which activities were excluded from the non-food-related responsibilities variable, enabling a meaningful sensitivity analysis.

# Supplemental Table 4. Average marginal association of time in responsibilities on time in food preparation: Alternate definition of responsibilities^†^

|  |  |  | Household composition subgroups | | | | | |
| --- | --- | --- | --- | --- | --- | --- | --- | --- |
|  | 1 Unadjusted | 2 Fully adjusted | 3 No children | 4 Children 0-4 | 5 Children 5-12 | 6 Children 13-17 | 7 Dual-parent households | 8 Single-headed households |
| Responsibilities | -0.055*** | -0.042*** | -0.038*** | -0.059*** | -0.066*** | -0.077*** | -0.071*** | -0.048*** |
| SE | (0.003) | (0.003) | (0.003) | (0.008) | (0.006) | (0.008) | (0.006) | (0.010) |
| n | 37368 | 35895 | 24247 | 4115 | 6493 | 3723 | 8104 | 2284 |

*** p<.001, ** p<.01, * p<.05

^†^ Marginal associations estimated by double hurdle models with robust standard errors clustered on state of residence. Time diary days that were holidays were excluded. Models include adults (≥18 years) who reported being primarily or partially responsible for meal preparation in the household. Time spent in responsibilities was modeled in the selection model and in the linear model. Control variables were the same in the selection and linear models and included respondent sex, age, age^2^, race/ethnicity, educational attainment, whether the respondent was married, poverty-income ratio, SNAP participation, employment status, usual number of hours worked per week, household size, indicators for household children ages 0-4 years, 5-12 years, and 13-17 years, and an indicator for single-parent households. Models 7 and 8 did not include a control variable for single-parent households. Models 2-8 included fixed effects for diary day of the week, survey year, and state of residence.

# Supplemental Table 5. Evaluating the importance of unobserved variables^†^

|  | Food preparation | |
| --- | --- | --- |
|  | Without controls | With controls |
| Responsibilities | -0.045***  (0.002) | -0.036***  (0.002) |
| R-squared | 0.023 | 0.146 |
| Oster bounds |  | [-0.031 – -1.150] |
| δ |  | 3.472 |

**p* < 0.05, ***p* < 0.01, ****p* < 0.001.

^†^ Estimates from OLS models with robust standard errors clustered on states. Fixed effects for state were included in models with and without controls. The full model controls for respondent sex, age, age^2^, whether the respondent is married, educational attainment, household poverty-income ratio, employment status, number of hours worked, the age category of the youngest child, diary day of the week, survey year, and state of residence.
